# Optimal integration of intraneural somatosensory feedback with visual information: a single-case study

**DOI:** 10.1038/s41598-019-43815-1

**Published:** 2019-05-27

**Authors:** G. Risso, G. Valle, F. Iberite, I. Strauss, T. Stieglitz, M. Controzzi, F. Clemente, G. Granata, P. M. Rossini, S. Micera, G. Baud-Bovy

**Affiliations:** 1 Robotics, Brain and Cognitive Sciences (RBCS), Istituto Italiano di Tecnologia, Genoa, Italy; 2 DIBRIS, Università degli studi di Genova, Genoa, Italy; 3 Bertarelli Foundation Chair in Translational Neuroengineering, Centre for Neuroprosthetics and Institute of Bioengineering, School of Engineering, École Polytechnique Fédérale de Lausanne (EPFL), Lausanne, Switzerland; 4 The Biorobotics Institute, Scuola Superiore Sant’Anna, Pisa, Italy; 5 Institute of Neurology, Catholic University of The Sacred Heart, Policlinic A. Gemelli Foundation, Roma, Italy; 6 Laboratory for Biomedical Microtechnology, Department of Microsystems Engineering–IMTEK & Bernstein Center, University of Freiburg, Freiburg, D-79110 Germany; 7 Vita-Salute San Raffaele University & Unit of Experimental Psychology, Division of Neuroscience, IRCCS San Raffaele Scientific Institute, Milan, Italy

**Keywords:** Perception, Brain-machine interface

## Abstract

Providing somatosensory feedback to amputees is a long-standing objective in prosthesis research. Recently, implantable neural interfaces have yielded promising results in this direction. There is now considerable evidence that the nervous system integrates redundant signals optimally, weighting each signal according to its reliability. One question of interest is whether artificial sensory feedback is combined with other sensory information in a natural manner. In this single-case study, we show that an amputee with a bidirectional prosthesis integrated artificial somatosensory feedback and blurred visual information in a statistically optimal fashion when estimating the size of a hand-held object. The patient controlled the opening and closing of the prosthetic hand through surface electromyography, and received intraneural stimulation proportional to the object’s size in the ulnar nerve when closing the robotic hand on the object. The intraneural stimulation elicited a vibration sensation in the phantom hand that substituted the missing haptic feedback. This result indicates that sensory substitution based on intraneural feedback can be integrated with visual feedback and make way for a promising method to investigate multimodal integration processes.

## Introduction

Nowadays there is considerable evidence that the brain integrates redundant information, routinely and unconsciously, in a statistically optimal manner^1–5^. The optimal integration hypothesis prescribes that the best estimate concerning the attribute of interest corresponds to a weighted average between the cues^3^:

1 $${s}_{VH}={w}_{V}{s}_{V}+{w}_{H}{s}_{H}$$

where *S*_*V*_ and *S*_*H*_ are the visual and haptic size cues. The weights *W*_V_ and *W*_*H*_ (*W*_V_ + *W*_H_ = 1) should be proportional to the reliability of the stimulus:

2 $$\begin{array}{cc}{w}_{V}=\frac{{R}_{V}}{{R}_{V}+{R}_{H}} & {w}_{H}=\frac{{R}_{H}}{{R}_{V}+{R}_{H}}\end{array}$$

where the reliability *R*_i_ = 1/${\sigma }_{i}^{2}$ is simply the inverse of the noise (variance) of the corresponding cue. Assuming that the visual and haptic cues are independent and normally distributed, it can be shown that the optimal (or Maximum Likelihood) estimate *s*_*VH*_ that combines the two cues has a variance:

3 $${\sigma }_{VH}^{2}=\frac{{\sigma }_{V}^{2}{\sigma }_{H}^{2}}{{\sigma }_{V}^{2}+{\sigma }_{H}^{2}}$$

that is not only lower than that of the single cues but the lowest possible given the noise associated with each cue. It is easy to show that the improvement brought by the integration is maximal when the two sensory modalities are equally reliable^3,5^. The noise associated with each channel can be determined in behavioral experiments by measuring the Just-Noticeable-Difference (JND), i.e. the smallest difference between two stimuli that can be discriminated reliably.

Recently, implantable neural interfaces have yielded promising results in restoring haptic sensation to the amputee. Both invasive and non-invasive methods have been developed to restore sensory information to prosthesis users in the last decade^6–11^. In particular, various types of neuroprostheses have been developed to recover lost functionalities at different levels of the nervous system^12,13^, including the restoration of peripheral sensory feedback thanks to bidirectional upper limb prostheses^8,10,14–16^. Several studies have shown that peripheral nerve stimulation through neural interfaces can restore somatotopic and modality-matched sensations^8,11^ showing improvements in prosthesis control^17,18^ and embodiment^19,20^ even in long term applications^17,21^. Sensory feedback delivered by these new neural interfaces can be used to identify the stiffness and shape of objects^10^, to discriminate the spatial coarseness of texture surfaces^15^ and to improve prosthesis control^11,21^.

One key challenge for the adoption and commercialization of recent multifunction upper limb prostheses is the integration of the sensory feedback from different sensory modalities^22^. Often during everyday life, prosthesis users’ ability to regulate their actions depends on multisensory stimuli such as the audible noise from the motor, pressure information from the socket or visual information about the completion of a movement, which require attention from the user unlike multi-sensory feedback normally used in hand control^23–25^.

The objective of this study is to test whether intraneural stimulation substituting natural haptic feedback is integrated optimally with visual feedback. To that end, we adapted the size discrimination task used by Ernst and Banks^3^ and prepared a set of cylinders that fitted the prosthetic hand well (see Methods). The task consisted in judging which of two hand-held cylinders appeared larger. The participant wore a headset with white noise to suppress audio cues and custom eyeglass to blur vision of the cylinder.

Information about the object size was provided visually and/or by stimulating electrically the nerve structures still present in the amputated arm. Specifically, we used a sensory substitution approach based on intraneural stimulation of the ulnar nerve with TIME electrodes^10,26^ to provide a somatosensory cue about the object size (see Methods). The somatosensory feedback was triggered by the contact of the robotic hand with the object and lasted until the object was released. The amplitude of the intraneural stimulation was proportional to the object size and thus corresponded to robotic hand aperture for the duration of the grasp.

Before starting the experiment, we characterized the sensations evoked by the neural stimulation (see ‘Somatosensory stimulus physical and perceptual characterization’ section). Perceptually, the stimulus felt as a vibration of the ulnar side of the phantom hand. The patient did not report any delay between the somatosensory and visual feedback when grasping an object. We adjusted the intensity of the neural stimulation to obtain a somatosensory discrimination threshold similar to those obtained by healthy subjects^27^. We also adjusted the level of blur to obtain a visual discrimination threshold similar to the somatosensory discrimination threshold in order to maximize the margin of improvement predicted by the optimal integration model in the bimodal conditions^3,5^.

The participant underwent a brief learning session (<10 min) to help map the stimulation intensity to the object size. We first instructed the participant to explore the new information by looking at the robotic limb while it was passively opened and closed. Then, we turned the control on and instructed the participant to actively explore the environment, grasping various objects and performing opening and closing movement with the prosthesis. The participant quickly expressed confidence in interpreting the sensation, as well as a readiness to initiate the trials.

**Figure 1:**
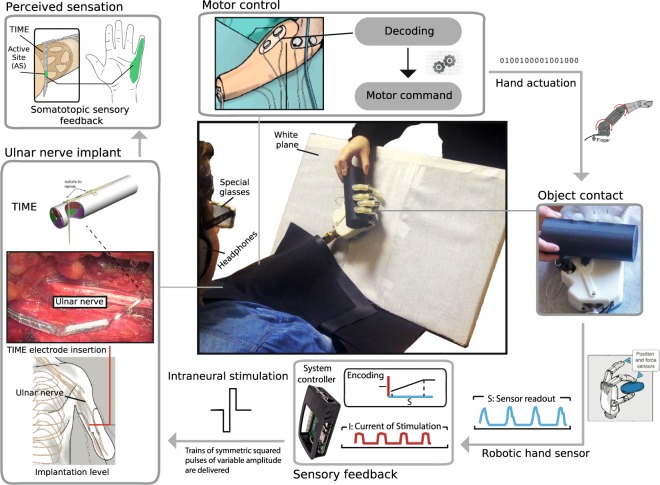
Experimental setup. The figure shows the white plane which the robotic hand leans on. The patient wore special glasses to degrade her vision and headphones to hear white noise during the experiment. The robotic hand is controlled using sEMG activity acquired from the subject’s dorsal and ventral forearm muscles, and classified into distinct motor commands (top). As the robotic hand closes its fingers around the object, both force and position are measured in real time (right). Information about force and position is then used to deliver stimulation pulses (somatosensory feedback; bottom). Somatosensory perception is provided by eliciting a sensation that corresponds to the finger and palm that are touched (top left). The sensory stream is delivered by using intraneural stimulation through TIME electrodes implanted in the proximal part of the ulnar nerve (left). The TIME implant is inserted transversally through the exposed nerve fascicles.

In the experiment, each trial consisted in the presentation of two cylinders to the participant by placing them in the robotic hand successively (Fig. 1). The diameter of one cylinder, i.e. the *comparison stimulus*, varied from trial to trial (between 55 and 65 mm) while the diameter of the other cylinder, i.e. the *standard stimulus*, did not vary across trials (60 mm unless a conflict was introduced between the sensory cues, see below). During each presentation, the participant closed and reopened the hand with the cylinder using a voluntary EMG command. At the end of each trial, the participant was asked to indicate which cylinder felt larger (two‐interval forced‐choice procedure).

The experiment included two unimodal and three bimodal conditions. In the *unimodal somatosensory* condition, the participant closed her eyes during the whole trial while the information about the cylinders’ size was conveyed by neural stimulation of the ulnar nerve. In the *unimodal visual condition*, the participant did the same with eyes open, and the ulnar nerve was not stimulated. In the *bimodal conditions*, visual and somatosensory information was provided simultaneously and could be congruent or not. The somatosensory and visual cues of the standard stimulus indicated a 60 mm cylinder diameter in the congruent condition or slightly conflicting diameters in the incongruent conditions (60 ± 2 mm). The comparison stimulus always provided congruent visual and somatosensory information.

The responses of the participant in each condition were fitted with a cumulative normal probability distribution using maximum likelihood estimation to obtain a psychometric function representing the probability of judging the comparison stimulus as larger than the standard stimulus. For each psychometric curve, we computed the point of subjective equality (PSE), i.e. the cylinder diameter that was perceived as larger than the standard in 50% of the trials. We also computed the discrimination threshold ‐ or Just Noticeable Difference (JND) – as the difference between the PSE and the cylinder diameter that is perceived to be larger than the standard in 84% of the trials (see methods for more details).

The results in the bimodal conditions were compared to the predictions derived from results in the unimodal conditions and the optimal integration hypothesis. In particular, the JND in the bimodal condition was lower than the visual and somatosensory JNDs as predicted by Eq. 3. We also verified that the PSEs in the bimodal conditions corresponded to the values predicted by Eq. 1.

## Results

**Figure 2:**
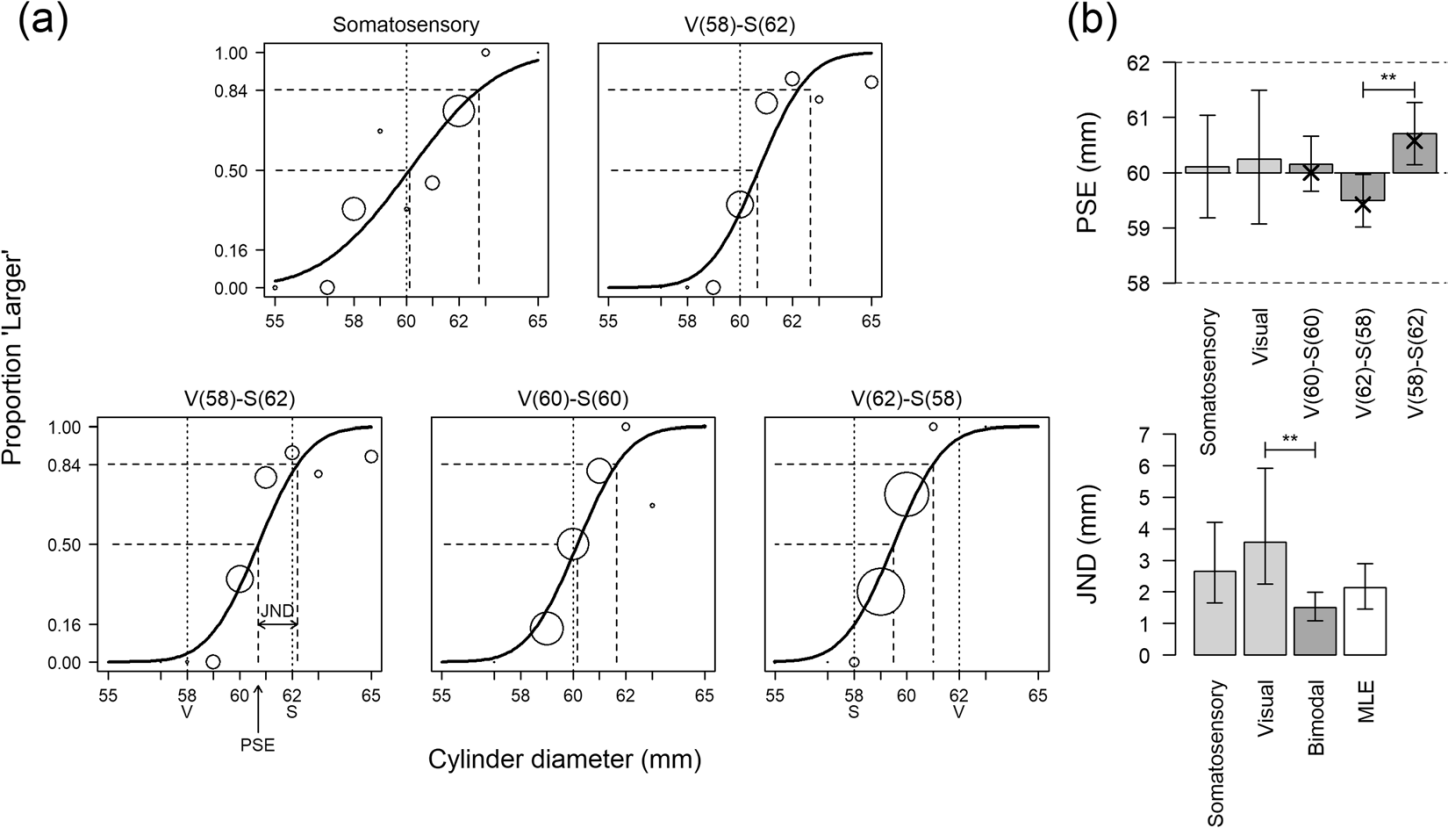
Panel (a) Psychometric curves in the unimodal (*top row*) and bimodal (*bottom row*) conditions. The x-axis represents the diameter of the comparison stimulus (in mm). The y-axis corresponds to the proportion of responses in which the comparison stimulus was larger than the standard. In the bimodal *congruent* condition V(60)-S(60), the visual and somatosensory characteristics of the standard corresponded to a 60 mm diameter cylinder (*vertical dotted line*). In the bimodal *incongruent* conditions V(58)-S(62) and V(62)-S(58), the visual and somatosensory characteristics of the standard stimulus differed by 4 mm (*vertical dotted lines*). The somatosensory and visual characteristics of the comparison stimulus were congruent in all bimodal conditions. The *vertical dashed line* that corresponds to a proportion of 50% of ‘Larger’ responses indicates the Point of Subjective Equality (PSE) (see, for example, arrow in V(58)-S(62) panel). The *distance between the two vertical dashed lines* corresponds to the Just Noticeable Difference (JND), which is the difference between the PSE and the cylinder diameter that is perceived to be larger than the standard in 84% of the trials. Panel (b) Representations of PSEs (*top)* and JNDs (*bottom)* for unimodal and bimodal conditions. The *crosses* in the top panel indicate the biases predicted by the optimal integration model. The *white bar* in the bottom panel represents the JND predicted by the optimal integration model for the bimodal conditions. The crosses in the PSE plot correspond to the predicted biases. The *vertical bars* correspond to the 95% bootstrap confidence interval (N = 5000 bootstrap samples). *Horizontal bars* denote statistically significant differences (False Discovery Rate adjusted bootstrap p-value **p < 0.01).

Figure 2a shows the probability of judging the comparison stimulus to be larger than the standard in the two unimodal conditions (top row). The goodness-of-fit tests for all psychometric functions were not statistically significant (p > 0.91), indicating a good fit with the data. The Point of Subjective Equality (PSE) indicates the cylinder diameter that was judged equal to the 60 mm standard. As expected, the PSE was close to the standard in the unimodal conditions. The JND corresponds to the difference between the cylinder diameter that was judged larger than the standard in 84% of the trials and the PSE. Steeper curves corresponded to smaller JNDs. Assuming that the estimates of the cylinder size were normally distributed, this JND definition corresponded to one standard deviation. Figure 2b shows the PSEs and JNDs in each condition, together with the Maximum Likelihood Estimate (MLE) predictions.

The JND was smaller in the somatosensory condition than in the visual condition (2.7 ± 0.6 versus 3.6 ± 0.8 mm), indicating that the artificial somatosensory stimulation gave a more reliable (less variable) estimate of the cylinder diameter than the blurred vision. Therefore, following Eq. 2, the artificial somatosensory cue has an optimal weight of 64% while the visual cue has a weight of only 36%. In the bimodal conditions, the two sensory modalities conveyed simultaneous information about the cylinders’ size. In agreement with the optimal integration model, the JND was smaller in the bimodal condition (1.5 ± 0.2 mm) than in the visual (3.6 ± 0.8 mm) or somatosensory (2.7 ± 0.6 mm) condition. However, the difference was statistically significant with the visual JND (bootstrap mean difference: −2.1 ± 0.98, *p* = 0.002) and only close to significance with the somatosensory JND (**−**1.8 ± 0.7, p = 0.061). This observation is in agreement with the optimal integration model since the margin of improvement that optimal integration can provide is more limited when one sensory cue is more reliable than the other one. The bimodal JND did not differ from MLE JND (2.13 ± 0.2 mm) statistically (0.6 ± 0.4, p = 0.183).

The PSE in the unimodal condition**s** and in the congruent bimodal condition V(60)-S(60) were near the standard, indicating an absence of bias. When the sensory cues are not congruent, optimal integration principles predict that the PSE should be biased toward the most reliable sensory modality (see Eq. 1). In the incongruent conditions with a 4 mm conflict between the visual and artificial somatosensory information, we found that the difference between the PSEs of the two incongruent conditions V(58)-S(62) and V(62)-S(58) was statistically significant (bootstrap mean difference: 1.2 ± 0.4, p = 0.008). Accordingly, the top panel of Fig. 2b shows that the PSE was biased by 0.5–0.6 mm (see *crosses*) toward the somatosensory modality as predicted by the maximum likelihood estimate (MLE) model. In fact, the PSE predicted by MLE explains 99% of the variance of the PSE observed in the bimodal conditions. The patient never noticed the small conflict between the sensory cues of the standard stimulus in the incongruent bimodal conditions.

In order to find out whether the discrimination threshold varied during the experiment, we verified whether the probability of judging the comparison stimulus to be larger than the standard changed between the first and second half of the trials in each condition (see Data Analysis section). This test revealed no change in the somatosensory (p = 0.351), visual (p = 0.432) or bimodal (p = 0.748) conditions.

## Discussion

When grasping an object, visual, proprioceptive and tactile cues provide redundant information about its size^3^. Previous research has shown that the nervous system continuously reassesses the reliability of each sensory modality and that somatosensory and visual cues are optimally integrated. For example, it has been shown that visual reliability decreases with the amount of blur added to the visual cue^3,5^ or when the size of the object is difficult to assess visually because it is grasped along the axis of vision^28^.

In this study, we demonstrated that an amputee with a bidirectional prosthesis optimally integrated an artificial somatosensory feedback with vision. To that end, we adapted the object size discrimination task that had been used to demonstrate that adults integrate visual and artificial somatosensory information optimally in a seminal study^3^. Neural stimulation of the ulnar nerve substituted the haptic cues, which provide crucial information during object manipulation in healthy subjects^29,30^. The intensity of the stimulation directly encoded the size of the hand-held cylinder. The cylinder set and stimulation intensities were selected to mimic the haptic size discrimination ability of healthy subjects^3,27^. Like in previous studies^3,5,28^, vision was blurred so that the visual discrimination threshold would correspond to the somatosensory discrimination threshold. Our results clearly indicate that the participant optimally integrated information about the object’s size from the two sensory modalities. Evidence for integration comes from both the lower discrimination threshold and the shift of the PSE in the bimodal conditions.

The stimulation strategy used in this study differed from previous studies with this patient where the amplitude of the stimulation was proportional to the pressure applied on a surface (tactile information)^10,21,31,32^ and robotic hand closure^31^ (position information). In particular, a notable difference with D’Anna *et al*.^31^ is that they used two different stimulation channels for tactile and (remapped) proprioceptive feedback. The tactile feedback was referred to most of the ulnar innervation area and encoded the measured force applied by the prosthetic digit. The position feedback encoded robotic hand closure without interruption and was referred to the medial part of the forearm close to phantom wrist. Here, a single-channel encoding strategy substituted the natural touch sensation with a vibration sensation that was referred to a single site, the ulnar side of the phantom hand. Moreover, the stimulation started only when the robotic hand had completed the grasp and the prosthetic digit touched the object (tactile event). Finally, amplitude of modulation increased proportionally with the cylinder size and, thus, grasp aperture (positional information). Despite a limited training session and the fact that the stimulus did not correspond to a natural touch sensation, the patient discriminated object size stably during the experiment, as shown by the absence of a difference between early and late trials.

A few studies have started to examine multisensory integration with artificial sensory feedback, using both invasive and non-invasive methods. Dadarlat and colleagues^33^ found that non-human primates can combine an artificial multichannel intra-cortical micro-stimulation signal (ICMS) about hand position with vision to form an optimal estimate of hand movement direction. However, this study intentionally created unnatural mappings between the micro-stimulation and the direction of hand movement. In contrast with this study, the primates required a long training process to learn the associations between movement direction and the ICMSs. Another difference with Dadarlat *et al*.^33^ is that neural stimulation in our study occurred in a peripheral nerve rather than intra-cortically.

Marasco *et al*.^9^ used vibratory feedback to elicit complex illusory movement sensations and found that tactile and visual information were optimally integrated in a task where the amputee had to detect a temporal delay between the voluntary motor command that triggered the closure of the prosthetic hand and the beginning of the sensory feedback. In such a task, only the timing of the tactile and visual feedback is relevant^9^. In contrast, the neural stimulation in our size discrimination task conveyed magnitude information about the cylinder size. It remains to be seen whether vibratory and visual feedback can be used to elicit optimally integrated percepts that refer to the size or shape of hand-held objects.

This study presents several limitations and characteristics that should be investigated further. First, the results are based on a single patient and need to be confirmed with additional subjects.

Second, future research should aim at demonstrating optimal integration in more natural conditions. Since the relationship between the object size and the neural stimulus is essentially arbitrary, it might be possible to demonstrate optimal integration with artificial somatosensory stimulation levels that yield the same discrimination performance as non-blurred vision. Testing the optimal integration hypothesis under these conditions would involve somatosensory and bimodal discrimination thresholds that correspond to super-human performance levels.

In this respect, it should be noted that neural stimulation provided unambiguous and noiseless information about the cylinder’s size in our study because it was set directly by the experimenter as a function of object size. While object size might be measured by the sensors in the prosthesis when grasping the object, the estimated object size is likely to be less reliable for various reasons such as trial-to-trial variability in the placement of the fingers on the objects, etc. Obtaining reliable information about object size might become particularly difficult to achieve if there is little size difference between the objects. The somatosensory discrimination threshold might have a lower bound for technical and pragmatic reasons and future research should address this issue.

Third, previous research has shown that redundant information from different sensory modalities must be spatially and temporally correlated for integration to occur^28,34–36^. In this study, neural stimulation was triggered by the contact between the cylinder and the robotic hand when the patients voluntarily closed the hand via sEMG. The delay between the contact and the delivered stimulation was 20 ms, well within the integration window shown in previous studies^37^. It would be interesting to explore the extent to which active control of the prosthesis and simultaneity between visual and tactile feedback is important to foster integration in this context.

Finally, the sensations elicited by the neural stimulation were remapped to the phantom hand rather than perceived in-loco, preserving somatotopic information at a gross scale. However, these sensations differed in important ways from the sensations occurring when touching or grasping an object with the healthy hand. In a preliminary sensation characterization test, the participant reported a graduated sensation of vibration in the 5^th^ digit and ulnar part of the palm during the stimulation. Perceptually, object size was encoded by the intensity of the vibration felt on the phantom hand rather than by the perceived position of the fingers. Despite these differences, the artificial somatosensory sensation that substituted the natural haptic cue was integrated with the visual cue in an optimal fashion. Our results suggest that a natural sensation is not a necessary condition for optimal integration, which is an interesting observation in the on-going debate on stimulus features that determine whether sensory cues must be integrated or not^38^. Still, it would obviously be preferable that the artificial stimulus resemble more the natural sensation of touch when grasping an object. In this respect, it should be noted that major strides have been made recently towards the implementation of codes for neuroprosthetic devices to deliver rich, natural and versatile tactile sensations^32,39,40^. Compared to non-invasive methods, neural stimulation could in principle provide more specific and qualitatively diverse sensory feedback to the user. This biomimetic approach to sensory restoration, together with our findings and the development of increasingly sophisticated anthropomorphic robotic limbs opens encouraging scenarios toward the restoration of lost limb functioning.

## Methods

### Participant

The participant was a 53-year-old female trans-radial (proximal third of the forearm) amputee. The amputation occurred in December 2015, following a traumatic accident at work. In July 2017, the participant underwent implantation of TIME (transverse intrafascicular multichannel electrode)^26,41^ under general anesthesia through a 15 cm long skin incision to the left arm. Two TIMEs were placed in both the median and ulnar residual nerves^42^. Stimulation of site 12 (R5) of the TIME implanted in the ulnar nerve (implant TIME 3) induced the sensation of a gradual, charge-dependent vibration on a skin region of the 5^th^ finger. Ethical approval was obtained from the Institutional Ethics Committees of Policlinic Agostino Gemelli at the Catholic University, Rome, Italy, where the surgery was performed. The protocol was also approved by the Italian Ministry of Health. During the entire length of our study, all experiments were conducted in accordance with relevant guidelines and regulations. The participant signed an informed consent for all experiments and for publication of identifying information/images.

### Apparatus

#### Bidirectional hand setup

The participant was fitted with a customized bidirectional research prosthesis, allowing her to control the opening and closing of a prosthetic hand by processing surface electromyographic (sEMG) signals, and providing sensory feedback by means of electrical stimulation of the peripheral nerves. The robotic hand integrated tension force sensors within each digit except the last two fingers (IH2 Azzurra, Prensilia, Italy). The hand was controlled by a custom-build, multithreaded C++ software running on a RaspberryPi 3, Model B (RasperryPi foundation, UK) single board computer. A recording and stimulating device (Neural Interface Processor NIP, Ripple Grapevine, Ripple LLC, US) was also connected to the central single board computer, acquiring sEMG data from four bipolar channels and providing stimulation outputs to the four neural electrodes. A custom molded socket was built with integrated screws to easily fix the robotic hand on the end. Holes were drilled to allow for the placement of sEMG electrodes on the stump. Figure 1 schematically represents the bidirectional hand setup used for the visuo-haptic task.

#### Prosthesis control

For prosthesis control, a simple three-state K-Nearest Neighbour (k-NN) classifier with three classes (open, close and rest)^43^ was used. Four bipolar channels of surface EMG (sEMG) were acquired from residual forearm muscles, two from the dorsal (digit extensor, extrinsic muscle) and two from the ventral (digit flexor, extrinsic muscle) side of the forearm. These signals were recorded over the belly of the muscles. The sEMG data were acquired with a sampling frequency of 2 kHz, and filtered using an IIR filter with 4^th^ order Butterworth characteristics, between 15 and 375 Hz, as well as a notch filter to remove 50 Hz power hum including higher harmonics at 100 and 150 Hz. For the k-NN classifier, the waveform length (WL) was computed over a window of 100 ms for each channel and fed to the classifier every 100 ms. The WL controlled hand actuation speed which was updated every 100 ms and allowed for a gated-ramp control of the robotic hand movements, described by the following relation, already reported in^21^:

$${V}_{hand}=\{\,\begin{array}{lll}{V}_{hand}+Clas{s}_{output} & {\rm{when}} & Clas{s}_{output}=-\,1\,{\rm{or}}\,1\\ 0 & {\rm{when}} & Clas{s}_{output}=0\end{array}\,$$

where *Class*_*output*_ is the output of the classifier and *V*_*hand*_ is a number in the range between 0 and 511 that controls the velocity of the motors of the robotic hand.

The hand motor velocity increased or decreased by 0.297 deg/sec when the output indicated closing or opening respectively. Otherwise it remained constant. In this way the participant was able to close, open or keep the hand still by modulating the hand speed.

### Experimental procedure and conditions

The participant sat in front of a table wearing headphones with white noise and eyeglasses to blur vision. Following other studies^3,5^, vision was blurred to avoid exclusive reliance on the visual modality, which is predicted by optimal integration principles when one modality is much more reliable than another. In this study, the participant wore customized eyeglasses that blurred her view of the cylinder (see below). A tilted plane on the table supported the prosthetic hand. The plane was covered with white linen to make the hand and stimuli easily visible. The distance between the hand and the eye was 55 cm. The prosthesis lay in a natural position but was slightly separated from the arm under a cloth to avoid the transmission of possible mechanical cues while grasping the object.

During each trial, two cylinders were successively placed in the robotic hand by the experimenter. The diameter of one cylinder (standard stimulus) was always the same (60 mm) while the diameter of the other cylinder (comparison stimulus) ranged from 55 to 65 mm (55, 57, 58, 59, 60, 61, 62, 63, and 65 mm). Each cylinder was placed in the participant’s robotic hand by the experimenter. All cylinders were 3D printed with a variable density to have the same weight (138 g) and length (150 mm). The presentation order of the standard and comparison stimuli was randomized. At the end of each trial, the participant verbally reported which cylinder (first or second) had appeared larger.

The main experiment included two unimodal and three bimodal conditions. In the *unimodal conditions*, the stimuli were presented only in the visual or artificial somatosensory modality. In the *somatosensory condition*, the participant was asked to close her eyes during the trial. In the *visual condition*, no artificial somatosensory feedback was delivered when the participant opened/closed the hand. In the *bimodal conditions*, the two sensory cues associated with the standard stimulus could be congruent or incongruent. In the *congruent condition* V(60)-S(60), the artificial somatosensory stimulation intensity corresponded to the actual size of the standard stimulus (60 mm), which was visually seen. In the two *incongruent conditions*, the standard stimulus’ information on cylinder diameter conveyed somatosensory and visually was different. In one incongruent condition V(62)-S(58), the somatosensory stimulation corresponded to a diameter of 58 mm while the visual stimulation (i.e. the actual size of the cylinder placed in the robotic hand) was 62 mm. The opposite was true in the second incongruent condition V(58)-S(62). The information conveyed by the two sensory modalities was always congruent for the comparison stimulus.

For each condition, the value of the comparison stimulus at each trial was controlled by two QUEST procedures. The QUEST is an adaptive psychophysical procedure to compute thresholds efficiently^44^. The two QUESTs aimed at stimulus values that were judged larger than the standard in 30% and 70% of the trials, respectively in order to collect data near the inflection points of the psychometric curves and estimate the slope reliably^45^. The experimental condition changed randomly every five trials. At each trial, one of the two QUESTs of the current condition was randomly selected. A condition was completed when 30 trials were collected by each QUEST (i.e., at least 60 trials per condition).

### Somatosensory and visual stimulus characterization and calibration

Before the main experiment, we conducted preliminary experiments to characterize the artificial somatosensory stimulus and to calibrate the artificial somatosensory and visual stimuli to make them equally discriminable in order to be able to demonstrate optimal integration with respect to an alternative hypothesis that the most reliable modality dominates. If one cue is much more reliable than the other, optimal integration principles give a very large weight to this cue in the bimodal condition and the result cannot be distinguished from the best-modality dominance hypothesis.

#### Somatosensory stimulus physical and perceptual characterization

**Figure 3:**
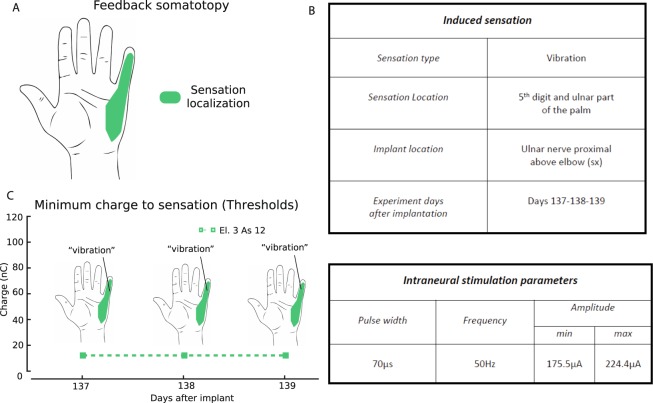
Physical and perceptual characteristics of the somatosensory stimulus. (**A**) Location on the phantom hand of the sensation elicited by the intraneural stimulation of the ulnar nerve by means of TIME electrode (somatosensory feedback somatotopy). (**B**) General information about the intraneural stimulation-induced sensations. The type of sensation quality is reported, as well as the sensation location and the implant location (top). Set of stimulation parameters used during the experiments referring to the electrode used (bottom). The experiments were performed with the same parameters in all sessions. The minimum charges needed to evoke a sensation, the location and the type perceived on the phantom hand are shown in (**C**) according to the days of interest.

The ability of the electrodes to elicit sensations by means of electric current stimulation was investigated before the experiment. To this aim, each of the 14 single contacts of each of the four TIME implants was stimulated with a train of cathodic-first, rectangular biphasic, charge-balanced pulses delivered by Ripple Grapevine LCC (Neural Interface Processor (NIP), Ripple Grapevine US). The frequency of the pulses delivered was 50 Hz for every trial. The injected electric charge varied within the safety limits indicated for the electrodes by the manufacturers and by the ethical committees. Elicited sensations reported by the participant (type, location, and strength on a scale from 1- barely perceivable, to 10 - painful pressure) were recorded (Fig. 3). The stimulation led to a vibration sensation on the 5^th^ digit and the ulnar part of the palm of the phantom hand. For the typical time scales involved in our experiments (order of minutes), the participant did not report relevant changes in sensation intensity, which would indicate the presence of adaptation. For all practical purposes, adaptation was insignificant during our experiments. Moreover, the sensation type, location and threshold of the artificial somatosensory sensory feedback were tested before starting the task and they remained stable and reliable over all three days of interest (Fig. 3). More information about the sensations elicited by all the active sites is provided in Petrini *et al*.^21^.

For the experiment, the artificial somatosensory stimuli corresponded to stimulation trains, which were injected when the robotic 5^th^ digit touched the object with a value related to the diameter of the cylinder (from 55 mm to 65 mm with 60 as reference). The intensity was determined in a preliminary phase so that the artificial somatosensory and visual stimuli would be equally discriminable. The safety limit was set to 120 nC^26^.

#### Somatosensory and visual stimulus calibration

First, we measured the ability of the participant to discriminate artificial somatosensory feedback produced by intraneural stimulation. Two stimuli were presented, and the participant’s task was to indicate which of the two was more intense. Each pair consisted of a standard charge (14 nC) and a comparison charge, selected in a comfortable range from 12 nC to 16 nC (step of 0.5 nC). The stimulation pulse-width was fixed at 70 µs. The experimental test comprised 90 trials. Each stimulus pair was presented 10 times, and both the order of the stimuli within the pair and the order of the pairs were randomized. The two pulse trains lasted 2 s and were separated by a 2 s inter-stimulus interval. The participant was instructed to focus on the intensity or magnitude of the sensation evoked. The participant was blinded to the particular stimulation conditions of each trial.

Second, we measured the ability of the participant to visually discriminate the cylinders’ size. As noted above, the participant wore eyeglasses to blur vision. Two cylinders, the standard stimulus (60 mm diameter) and one of the comparison stimuli, were successively presented by the experimenter at a distance of 55 cm in front a white background. The task was to indicate which of the two cylinders was larger. There were eight comparison stimuli with a diameter ranging from 55 to 65 mm, which were presented 7 times each. The order of presentation of the two cylinders within and among trials was randomized.

**Figure 4:**
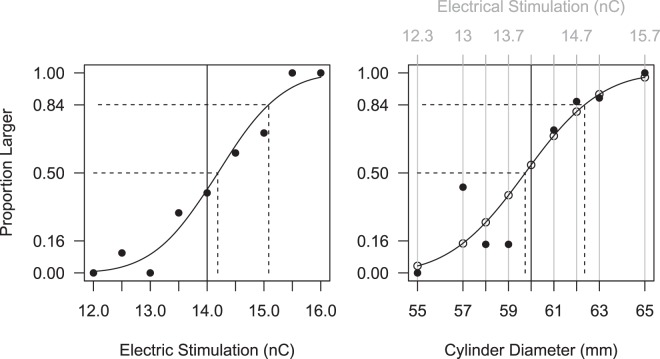
From cylinder diameter to neural stimulation. *Left:* Electrical stimulation discrimination experiment. On the x-axis, the electrical stimulation injected for the comparison stimulus (in nC). On the y-axis, the proportions of trials where the comparison stimulus (*horizontal axis*) appeared larger than the standard (*vertical solid line*). *Black dots* corresponds to the data points. Each stimulus was presented 10 times (N = 90). The psychometric function (*solid curve*) represent the probability of judging the comparison stimulus to be larger than the standard. The horizontal *distance between the two dashed lines* corresponds to the JND. *Right:* Mapping between the electrical stimulation and the stimuli size. The *grey scale* above the top margin indicates the charges used in the main experiment as artificial somatosensory stimuli. *Black dots* correspond to data points and *empty dots* indicate the probability of judging the comparison stimulus (*horizontal axis*) larger than the standard (*vertical line*) for the different stimulation levels (*grey scale*).

The participant’s responses were fitted with a cumulative Gaussian probability distribution to obtain a psychometric function representing the probability of judging the comparison stimulus to be larger than the standard stimulus (see Fig. 4). Then we computed the JND for the intraneural stimulation (*JND*_*ST*_) and for the visual stimulus (*JND*_*V*_; Fig. 4, left panel) from the results of these preliminary experiments. Finally, we computed the levels of intraneural stimulation intensity *I*(*s*_H_) that should be associated with the artificial somatosensory cue *s*_*H*_ to elicit somatosensory sensations that have the same discriminability as the visual stimuli (Fig. 4, right panel):

$$I({s}_{H})=({s}_{H}-60)\frac{JN{D}_{ST}}{JN{D}_{V}}+{I}_{60}$$

where *s*_*H*_ is the cylinder diameter in mm for the somatosensory modality, *JND*_*ST*_ = 0.897 nC and *JND*_*V*_ = 2.617 mm are the 84% discrimination thresholds for the neural stimulation and visual cue obtained in the preliminary experiments, and *I*_60_ = 14 nC is the intermediary level of stimulation that was selected to represent the standard stimulus. The neural stimulation intensities used during the visuo-haptic task were 12.28 nC (55 mm), 12.97 nC (57 mm), 13.31 nC (58 mm), 13.66 nC (59 mm), 14 nC (60 mm reference), 14.34 nC (61 mm), 14.69 nC (62 mm), 15.03 nC (63 mm) and 15.71 nC (65 mm). The stimulation train frequency was fixed at 50 Hz.

### Data analysis

The responses of the participant in each condition were fitted with a cumulative normal probability distribution using maximum likelihood estimation, i.e. with a Generalized Linear Model (GLM) with the comparison stimulus as predictor, Bernouilli error distribution and a probit link function to obtain a psychometric function representing the probability of judging the comparison stimulus as larger than the standard stimulus. Goodness of fit was assessed with Pearson’s χ^2^ normal goodness-of-fit test for generalized linear models.

The two unimodal conditions were fitted separately. To obtain a common slope estimate, the three bimodal conditions were fitted together, with a different intercept for each condition. For each psychometric curve, we computed the point of subjective equality (PSE), i.e. the cylinder diameter that was perceived as larger than the standard in 50% of the trials. We also computed the discrimination threshold - or Just Noticeable Difference (JND) – as the difference between the PSE and the cylinder diameter that is perceived to be larger than the standard in 84% of the trials. 84% JND corresponds to the standard deviation of the normal distribution underlying the psychometric function and is an estimate of the noise associated with the unimodal or bimodal cues. We computed the 95% confidence interval for the PSE and JND from 5000 parametric bootstrap samples with x fixed^46^. In the Results, we report the JND and the standard deviation of the bootstrap distribution.

The p-values for the pairwise difference in PSEs (or JNDs) between conditions were obtained by inverting the percentile bootstrap confidence intervals. Specifically, for each difference in PSEs (or JNDs), we computed the resampling distribution of the difference and the p value that corresponded to the largest equi-tailed confidence interval that excluded zero. We then adjusted this p-value for False Discovery Rate to account for the multiple comparisons involved in these pairwise tests. The difference between the optimal (MLE) JND and the bimodal JND was tested in a similar manner. We report the mean and standard deviation of the difference between the two bootstrap distributions.

To examine whether the performance improved during the experiment, we compared early and late trials by adding a predictor in the GLM models to indicate whether the trial belonged to the first or the second half of the condition. As before, the three bimodal conditions were fitted together with a common slope but a different intercept for each condition. We report the p value associated with this predictor.

## Data Availability

The datasets analyzed during the current study are available from the corresponding author on reasonable request.
